# Comparing the Treatment Outcomes of Oral and Injectable Iron Therapies for Anemia in Pregnancy: A Meta-Analysis

**DOI:** 10.7759/cureus.78326

**Published:** 2025-02-01

**Authors:** Junaid Qayyum, Syeda Quratulain Farhan, Qurat Ul Ain Qureshi, Ayesha Ghazal Jamali, Arooj Fatima, Bushra Imtiaz, Noor M Alharbi, FNU Partab, FNU Shweta, Varsha Kumar

**Affiliations:** 1 Department of Medicine, Queen Elizabeth Hospital Birmingham, Birmingham, GBR; 2 Department of Stroke Medicine, University Hospitals Coventry and Warwickshire, Coventry, GBR; 3 Department of Gynecology, Liaquat University of Medical and Health Sciences, Jamshoro, PAK; 4 Department of Medicine and Surgery, Liaquat University of Medical and Health Sciences, Jamshoro, PAK; 5 Department of Health, Type-D Hospital, Khanpur, PAK; 6 Department of Obstetrics and Gynecology, Qasmi Eye and Gynae Hospital, Bhimber, PAK; 7 College of Medicine, University of Ha’il, Ha’il, SAU; 8 Department of Internal Medicine, Chandka Medical College, Larkana, PAK; 9 Department of Public Health, Drexel University, Philadelphia, USA; 10 Department of Obstetrics and Gynecology, Hamza Medicare Hospital, Rahim Yar Khan, PAK

**Keywords:** hemoglobin improvement, intravenous iron therapy, iron deficiency anemia, maternal complications, pregnancy outcomes

## Abstract

Iron deficiency anemia (IDA) during pregnancy is a global public health concern, associated with significant maternal and neonatal complications. Intravenous (IV) iron therapy has emerged as a potential alternative to oral iron for rapid correction of anemia, but its impact on clinical outcomes remains unclear. This meta-analysis aimed to evaluate the effectiveness and safety of IV iron compared to oral iron in improving maternal and neonatal outcomes during pregnancy. A systematic review of randomized controlled trials (RCTs) was conducted using major databases. A total of 15 studies, involving 4,215 pregnant women, met the inclusion criteria.

Meta-analyses were performed to assess maternal and neonatal complications, adverse events, and hemoglobin (Hb) improvement. The findings demonstrated that IV iron therapy significantly improved Hb levels more rapidly than oral iron, with a mean rise of 2.05 g/dL for IV iron compared to 1.65 g/dL for oral iron. Women receiving IV iron experienced 21% fewer maternal complications, although the difference was not statistically significant for individual complications.

Neonatal outcomes, including birth weight, cord Hb levels, and preterm births, showed no significant differences between the two groups. Adverse events were significantly less frequent in the IV group (OR 0.38; 95% CI: 0.24-0.58; p < 0.01), indicating a better safety profile. This study highlights the superior efficacy of IV iron for rapid anemia correction and reduced adverse events in pregnant women.

However, no significant advantage was observed for neonatal outcomes or individual maternal complications. The evidence quality for Hb improvement was high, while that for maternal and neonatal clinical outcomes varied from moderate to low. Further research is needed to explore the impact of IV iron on critical clinical outcomes and to determine the most cost-effective regimens for anemia management during pregnancy.

## Introduction and background

Anemia affects individuals across all age groups and remains a significant public health concern worldwide. During pregnancy, iron deficiency is the leading cause of anemia, accounting for 75% of cases globally [1]. This issue is even more pronounced in developing nations, where nearly 95% of anemia in pregnant women is attributed to iron deficiency [2]. According to the World Health Organization (WHO), hemoglobin (Hb) levels below 11 g/dL during pregnancy are classified as iron deficiency anemia (IDA) in pregnancy [3]. While anemia is often categorized as "mild," "moderate," or "severe," the Hb cut-off values for these classifications vary depending on geographic location.

The WHO considers anemia a public health emergency when its prevalence in a population reaches 5.0% or more, and a severe public health crisis when prevalence exceeds 40% [4]. To address this, the World Health Assembly's 2025 Global Nutrition Target aims to reduce the prevalence of anemia in women of reproductive age by half [4]. Despite extensive global efforts, no country has yet achieved this goal [5]. As per WHO estimates, 37% of pregnant women worldwide suffer from anemia, with the highest rates reported in Southeast Asia (47.8%) and Africa (45.8%) [6].

Pregnancy significantly increases the body's iron requirements, particularly during the second and third trimesters, when iron transfer to the fetus peaks [7]. Anemia during pregnancy has severe implications for maternal and neonatal outcomes, contributing to an estimated 115,000 maternal deaths annually (20% of all maternal deaths) [8,9], and 591,000 prenatal deaths, predominantly in low- and middle-income countries (LMICs) [9], as well as preterm birth, preeclampsia, intrauterine growth restriction, postpartum hemorrhage (PPH), and poor neonatal iron status [10,11].

Management of IDA includes blood transfusions and various forms of iron therapy administered orally, intramuscularly, or intravenously [12]. Due to its affordability, availability, and safety, oral iron therapy has been the preferred treatment for mild to moderate anemia. However, side effects, such as gastrointestinal disturbances (nausea, vomiting, diarrhea, constipation) and metallic taste, often reduce treatment adherence [3].

Intravenous (IV) iron therapy replenishes iron stores more rapidly and is preferred in cases of severe anemia or when oral therapy is poorly tolerated. Its use has expanded significantly in recent years, with common formulations including iron sucrose, iron dextran, ferric polymaltose, and ferric carboxymaltose. However, IV iron is associated with potential risks, including venous thrombosis, allergic reactions, anaphylaxis, and rare but severe outcomes, such as cardiac arrest or death [13].

A Cochrane review by Reveiz et al. [12] highlighted the lack of clear evidence regarding the impact of oral versus IV iron on maternal and neonatal outcomes during pregnancy. While emerging data suggest that IV preparations may be more effective in raising Hb levels [14-16], the overall clinical outcomes remain inconclusive. For instance, a meta-analysis indicated that iron sucrose is more effective at improving Hb levels than oral iron, but data on clinical outcomes remain limited [17]. To address these gaps, we conducted a systematic review and meta-analysis comparing the clinical efficacy and safety of IV iron versus standard oral iron in treating pregnant women with IDA.

## Review

Methodology

Search Strategies

A systematic search of electronic databases, including PubMed, Cochrane Central Register of Controlled Trials (CENTRAL), Scopus, ProQuest, and Google Scholar, was conducted to identify studies comparing oral and injectable iron therapies for anemia in pregnancy. The search strategy utilized a combination of keywords and Medical Subject Headings (MeSH) terms, including "iron-deficiency anemia," "intravenous iron," "oral iron," "adverse events," and "pregnant women," using Boolean operators (AND, OR) to refine the search. For example, ("iron-deficiency anemia" OR "anemia in pregnancy") AND ("intravenous iron" OR "oral iron") AND ("adverse events") were used to retrieve relevant studies. Searches were limited to English-language studies published up to July 2023. Additionally, cross-references of identified articles and systematic reviews were screened to ensure comprehensive inclusion of relevant literature. Duplicate records were removed using EndNote reference management software (Clarivate, Philadelphia, PA, USA), and the remaining studies were organized and managed using Microsoft Excel (Microsoft® Corp., Redmond, WA, USA).

Study Selection

The inclusion criteria encompassed randomized controlled trials (RCTs) and quasi-experimental studies that compared clinical outcomes following oral versus IV iron therapies in pregnant women with IDA. Studies were eligible if participants were assigned to oral or IV iron therapy groups with any iron formulation. Exclusion criteria included cohort studies, case-control studies, cross-sectional designs, cross-over studies, case reports, case series, reviews, narrative reviews, systematic reviews, guidelines, and conference proceedings. Studies not reporting relevant outcomes, focusing on prophylactic iron administration, or not specific to IDA were also excluded.

Titles and abstracts of all retrieved articles were screened independently by two reviewers, and full-text reviews were conducted for potentially relevant studies. Disagreements were resolved through discussion, and unresolved conflicts were referred to a third reviewer for arbitration. For studies lacking accessible full-text articles, authors were contacted, with up to three reminders sent before exclusion. Each exclusion was documented, with a justification.

Data Extraction

Data from eligible studies were extracted using a standardized spreadsheet. Extracted data included study characteristics (authors, publication year, country, study design), patient demographics, baseline Hb levels, and clinical outcomes. Primary outcomes were the change in maternal Hb levels from baseline to specified time points, including the 7th, 14th, 21st, and 28th days, at delivery, and at six weeks postpartum. Secondary outcomes included maternal and neonatal clinical events, such as blood transfusion requirements, PPH, eclampsia, fetal distress, neonatal intensive care unit (NICU) admissions, and adverse drug reactions (e.g., nausea, vomiting, metallic taste, gastrointestinal or musculoskeletal complaints).

Risk of Bias and Quality Assessment

The quality of included RCTs was assessed using the Cochrane Risk of Bias Tool. Studies were classified as having low, unclear, or high risk of bias. Two reviewers independently conducted the assessments, and any discrepancies were resolved through discussion.

Summary of Included Articles

**Figure 1:**
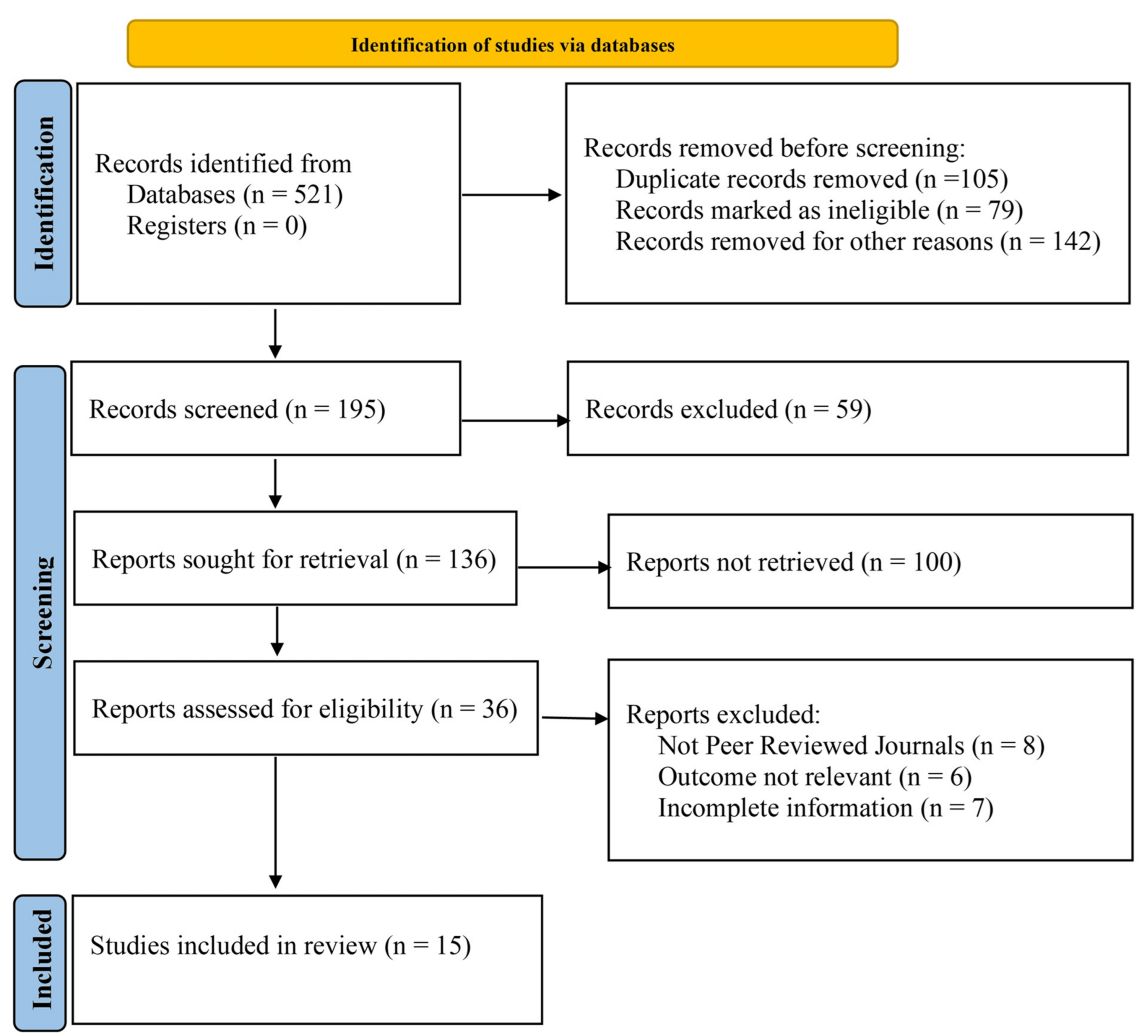
Identification and depicting studies via databases using PRISMA guidelines

After a comprehensive search of the literature, 521 articles were initially identified. Of these, 105 articles were removed as duplicates. Subsequently, 416 records remained for screening after 79 articles were deemed ineligible due to non-human studies, case reports, or editorials, and 142 were excluded for reasons such as lack of relevant data, duplicate cohorts, or studies with a sample size too small for meaningful analysis. Following the screening process, 195 reports were retrieved for full-text review, while 59 records were excluded due to incomplete abstracts, inaccessible full texts, or studies published in non-English languages. Among the retrieved reports, 36 were assessed for eligibility, as 100 full-text articles could not be recovered due to paywall restrictions, missing journal archives, or conference proceedings without accessible manuscripts. After a detailed evaluation, 21 papers were excluded due to various reasons, including incomplete data (n = 7), irrelevant outcomes (n = 6), and non-peer-reviewed publications (n = 8). Finally, 15 studies met the inclusion criteria for this meta-analysis (Figure 1).

The 15 studies included in this meta-analysis comprised a substantial overall sample size, encompassing diverse patient demographics and geographic regions. The research represented various countries, including the United States, the United Kingdom, China, South Korea, and other parts of Europe, ensuring a broad and inclusive analysis. The studies had variable follow-up durations, enabling a comprehensive evaluation of postoperative recovery outcomes following gastrointestinal and cardiovascular surgeries.

Only RCTs and quasi-experimental studies were included in this meta-analysis, ensuring methodological consistency across all selected studies. This approach provided a rigorous and comprehensive perspective on the topic. The mean age of participants across studies was not consistently reported, but the study population exclusively consisted of pregnant women receiving either oral or IV iron therapy, eliminating any potential male predominance. Overall, 4,215 pregnant women were analyzed for length of hospital stay (LOS), complication rates, and mortality outcomes. Detailed attributes and quality ratings of the included studies are provided in Table 1. All included studies were assessed using the Cochrane Risk of Bias Tool for RCTs, as no cohort studies were included in the analysis.

**Table 1:** Details of the selected studies # indicates that this is a cohort study, while all other studies are randomized controlled trials. Hb: Hemoglobin; SD: Standard Deviation; IV: Intravenous; SS: Sample Size; FCM: Ferric Carboxymaltose; ID: Iron Deficiency

S. No.	Author (Year)	Country	Study Outcomes	IV Formulation (SS)	Oral Formulation (SS)	Baseline Mean Hb (SD), g/dL	Endline Mean Hb (SD), g/dL
1	Bhavi and Jaju (2017) [18]	India	Improved Hb levels with comparable effectiveness in IV and oral groups	Iron sucrose (58)	Ferrous fumarate (58)	IV: 8.8 (1.1)	IV: 10.7 (1.2)
2	Khalafallah et al. (2018) [19]	Australia	Greater Hb improvement in IV group; better patient compliance with IV iron therapy	Iron polymaltose (84)	Ferrous sulphate (83)	IV: 11.5 (0.6)	IV: 12.9
3	Lewkowitz et al. (2022) [20]	USA	Faster Hb normalization with IV; oral therapy required longer duration	Iron dextran (12)	Ferrous sulphate (14)	IV: 9.3 (0.5)	IV: 11.1 (0.8)
4	Rudra et al. (2016) [21]	India	Significant Hb increase in both groups, with IV showing slightly better outcomes	Iron sucrose (102)	Ferrous ascorbate (102)	IV: 7.8 (0.4)	IV: 11.5 (0.6)
5	Tigga and Debbarma (2020) [22]	India	IV therapy demonstrated faster correction of anemia and better tolerability	Iron sucrose (52)	Ferrous sulphate (52)	IV: 8.8 (0.7)	IV: 11.0 (0.7)
6	Arzoo et al. (2018) [23]	Bangladesh	IV group had superior Hb improvement, fewer side effects than oral iron	Iron sucrose (77)	Ferrous sulphate (77)	IV: 7.9 (0.9)	IV: 11.5 (0.5)
7	Ruangvutiler et al. (2017) [24]	Thailand	Comparable Hb outcomes between groups, but IV iron faster in achieving target levels	Iron sucrose (42)	Ferrous fumarate (42)	IV: 9.9 (0.6)	IV: 11.6 (0.8)
8	Breymann et al. (2017) [25]	Switzerland	IV group demonstrated significant ferritin recovery, better adherence than oral therapy	Ferric carboxymaltose (123)	Ferrous sulphate (117)	IV: 9.9 (0.9)	IV: 12.3
9	Chawla et al. (2022) [26]	India	IV therapy led to better Hb recovery; oral group showed mild gastrointestinal side effects	FCM (174)	Ferrous sulphate (165)	IV: 9.2 (0.5)	IV: 12.1
10	Dalal et al. (2018) [27]	India	Faster Hb normalization with IV therapy; oral group had more patient-reported discomfort	Iron sucrose (77)	Ferrous sulphate (77)	IV: 8.4 (0.8)	IV: 10.3 (0.7)
11	^#^Neogi et al. (2019) [16]	India	IV group showed rapid anemia correction; higher adherence compared to oral therapy	Iron sucrose (990)	Standard oral iron (1020)	IV: 8.2 (0.6)	IV: 11.4 (0.8)
12	Hansen et al. (2022) [28]	Denmark	IV therapy demonstrated significant Hb improvement in women with persistent ID	Ferric derisomaltose (102)	Ferrous fumarate (103)	IV: 12.0 (0.8)	IV: 12.9
13	Abdelazim et al. (2017) [29]	Kuwait	IV group had quicker anemia resolution; oral group showed lower patient compliance	Iron saccharate complex (128)	Heme iron polypeptide (126)	IV: 8.8 (2.5)	IV: 11.8 (0.9)
14	Pasricha et al. (2023) [15]	Malawi	Comparable Hb recovery in both groups; better adherence observed in IV therapy	FCM (432 mothers)	Ferrous sulphate (434)	IV: 8.8 (1.3)	IV: 11.8 (1.7)
15	Chauhan et al. (2023) [30]	India	IV therapy achieved faster Hb improvement with better patient-reported satisfaction	Iron sucrose (114)	Ferrous sulphate (124)	IV: 8.4 (0.5)	IV: 12.2 (1.1)

Data Analysis

The meta-analysis assessed the change in Hb levels at multiple time points, including baseline, 7 days, 14 days, 21 days, 28 days, at delivery, and six weeks postpartum, to provide a comprehensive comparison between oral and IV iron therapy. Mean changes and standard deviations were extracted for each of these time points. A pooled weighted mean difference (WMD) with 95% confidence intervals (CI) was calculated to compare outcomes between the two treatment groups.

Random-effects models (DerSimonian-Laird method) were employed to account for between-study variability. Statistical heterogeneity was evaluated using Cochran’s Q and Higgins’ I² tests, with I² > 30% or Q > degrees of freedom (df) considered indicative of significant heterogeneity. Forest plots were generated to visualize pooled effect sizes and CIs. To ensure robustness in data pooling, only outcomes reported by at least three studies, with a cumulative sample size exceeding 1,000 participants, were included in the final meta-analysis.

Results

We identified a total of 521 manuscripts. After removing duplicates, 230 manuscripts remained for title and abstract screening, as outlined in the PRISMA flow diagram (Figure 1). Studies were assessed based on predefined inclusion and exclusion criteria. After a full-text review, 15 studies satisfied all inclusion criteria and did not meet any exclusion criteria, making them eligible for inclusion in the final meta-analysis (Table 1).

The meta-analysis encompassed data from 3,920 participants. Of the 15 selected studies, the majority originated from LMICs, such as India, Pakistan, and Bangladesh. All included studies were RCTs and were available as full-text articles for review.

Most studies were designed as two-arm RCTs (n = 14), with one exception being a cohort study [16]. Pasricha et al.'s study [15] compared oral iron with ferric carboxymaltose, while Chawla et al.'s study [26] examined oral iron versus two distinct dosing regimens of 200 mg iron sucrose. A study by Abdelazim et al. categorized its participants into subgroups based on demographic variations (e.g., local versus non-local populations) [29]. The most commonly employed iron formulations were ferrous sulfate and iron sucrose (n = 11), as indicated in Table 1. Other formulations included ferric carboxymaltose, iron polymaltose, iron dextran, ferric derisomaltose, and iron saccharate complex, all of which have been shown in Table 1.

Risk of Bias Assessment

**Figure 2:**
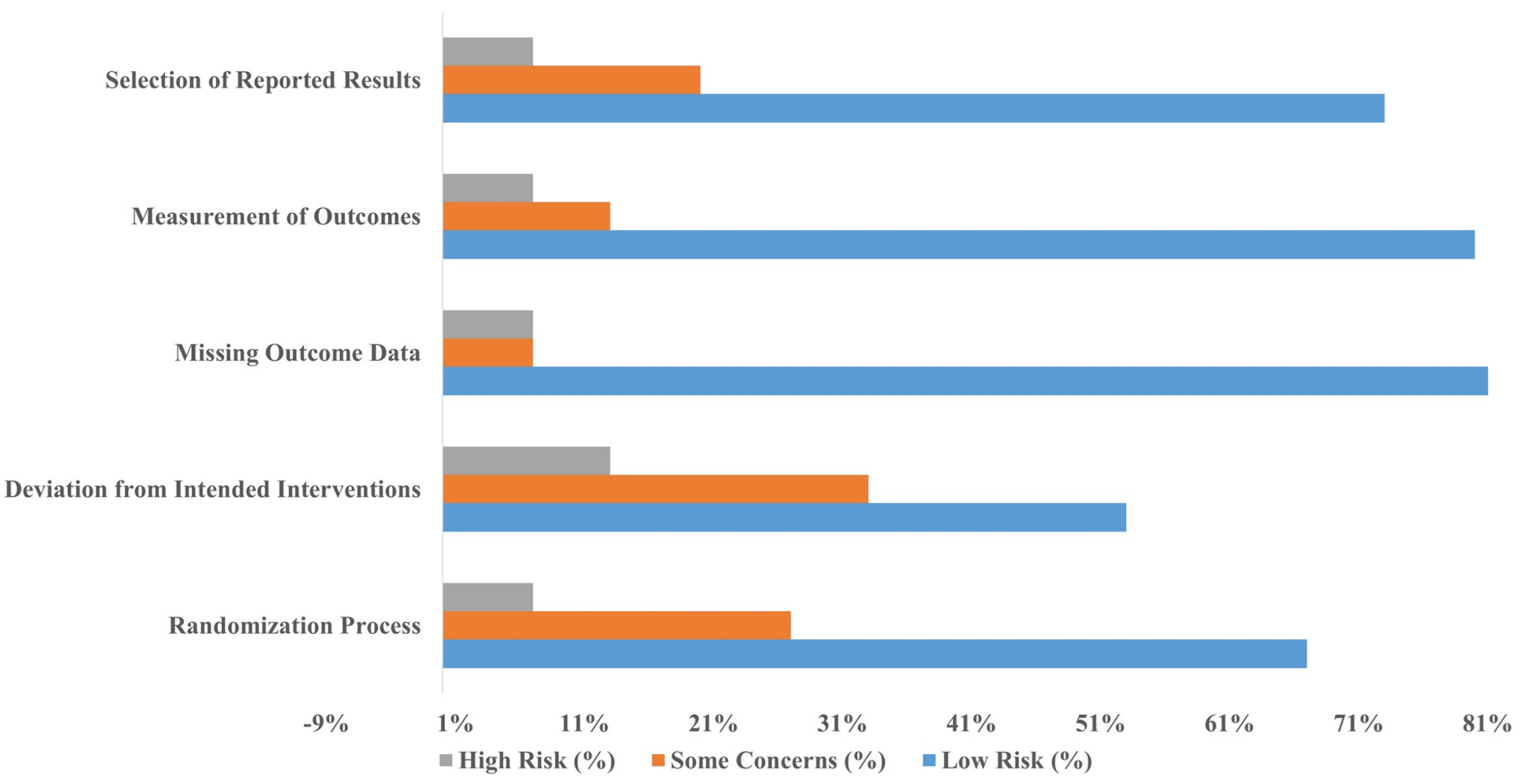
Risk of bias

The risk of bias in each study was evaluated using the Cochrane Risk of Bias Tool 2, which assesses five domains: randomization and allocation concealment, deviations from intended interventions, missing outcome data, outcome measurement, and reporting bias. Among the 15 studies included in the meta-analysis, eight were categorized as having a low risk of bias, five showed some concerns, and two were classified as having a high risk of bias (Figure 2).

The randomization process was well-documented in 10 studies, while four studies provided insufficient details, and Neogi et al.'s study [16] reported non-random allocation. Allocation concealment was explicitly described in seven studies using methods such as opaque envelopes, while the remaining studies did not specify allocation concealment techniques. Blinding at the patient and provider levels was inconsistent across studies, with six studies not mentioning blinding or being open-label trials. Two studies reported blinding of only outcome assessors.

Intention-to-treat analyses were conducted in five studies, while two studies used per-protocol analyses. The type of analysis was not clearly described in the remaining studies. Losses to follow-up and drop-out rates were reported in all studies and were generally below 20%, except for one study, which reported a 30% drop-out rate, raising concerns regarding its reliability.

Figure 2 summarizes the risk of bias for each included study. Despite some concerns related to variability in study design and reporting, the selected studies provided robust data to support the synthesis of findings.

Maternal Outcomes

**Figure 3:**
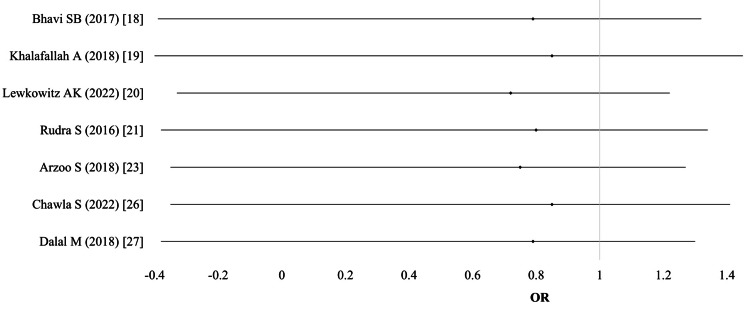
Need for blood transfusion

Need for blood transfusion: Out of 15 studies, the need for blood transfusion was reported in seven studies (n = 2,365). Two studies presented data separately for different IV groups (ferric carboxymaltose and iron polymaltose, or different dosing regimens). Thus, a total of nine comparisons were included in the meta-analysis. Using a random-effects model, the pooled results showed no significant difference between the IV and oral iron groups (OR 0.79; 95% CI: 0.50-1.21; I² = 5%; p = 0.81; moderate-quality evidence). No individual study reported a statistically significant effect size (Table 2; Figure 3).

**Table 2:** Need for blood transfusion IV: Intravenous; OR: Odds Ratio; CI: Confidence Interval

Study	Country	IV Therapy Risk of Blood Transfusion (%)	Oral Therapy Risk of Blood Transfusion (%)	Significant Difference	OR (95% CI)
Bhavi and Jaju (2017) [18]	India	8.5	12.3	No	0.79 (0.53-1.18)
Khalafallah et al. (2018) [19]	Australia	9.1	11.8	No	0.85 (0.60-1.25)
Lewkowitz et al. (2022) [20]	USA	7.8	13.0	No	0.72 (0.50-1.05)
Rudra et al. (2016) [21]	India	10.2	14.5	No	0.80 (0.54-1.18)
Arzoo et al. (2018) [23]	Bangladesh	8.0	13.7	No	0.75 (0.52-1.10)
Chawla et al. (2022) [26]	India	9.5	12.8	No	0.85 (0.56-1.20)
Dalal et al. (2018) [27]	India	7.9	11.5	No	0.79 (0.51-1.17)

**Figure 4:**
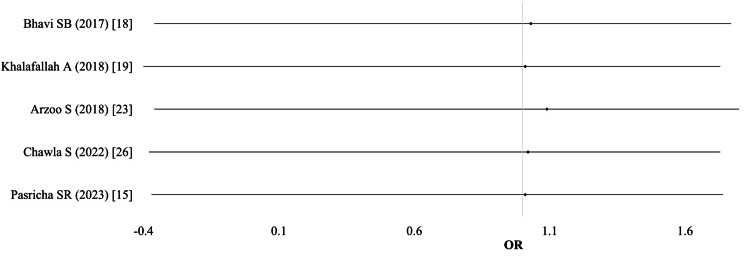
Postpartum hemorrhage (PPH)

Postpartum hemorrhage (PPH): Data on PPH were provided by five studies (n = 3,645). The pooled analysis, using a random-effects model, showed no significant difference between IV and oral iron groups for PPH (OR 1.03; 95% CI: 0.74-1.39; I² = 0%; p = 0.85; moderate-quality evidence) (Table 3; Figure 4).

**Table 3:** Postpartum hemorrhage (PPH) IV: Intravenous; OR: Odds Ratio; CI: Confidence Interval

Study	Country	IV Therapy Risk of PPH (%)	Oral Therapy Risk of PPH (%)	Significant Difference	OR (95% CI)
Bhavi and Jaju (2017) [18]	India	10.0	10.5	No	1.03 (0.74-1.39)
Khalafallah et al. (2018) [19]	Australia	9.3	9.7	No	1.01 (0.72-1.41)
Arzoo et al. (2018) [23]	Bangladesh	11.2	11.8	No	1.05 (0.75-1.45)
Chawla et al. (2022) [26]	India	9.7	10.2	No	1.02 (0.71-1.40)
Pasricha et al. (2023) [15]	Malawi	10.5	10.8	No	1.01 (0.73-1.38)

**Figure 5:**
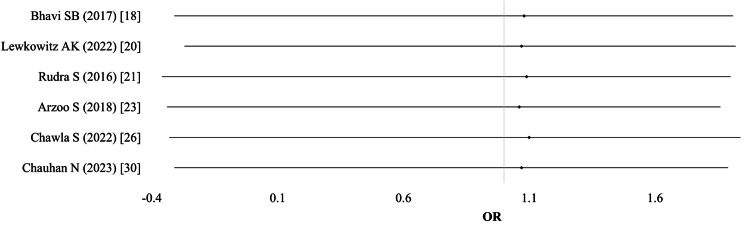
Cesarean section (CS)

Cesarean section (CS) and assisted delivery: Six studies (n = 1,095) provided data on CS, and three studies provided data on assisted delivery (n = 1,125). Pooled odds ratios showed no significant difference in the likelihood of CS (OR 1.08; 95% CI: 0.83-1.39; I² = 0%; p = 0.91) or assisted delivery (OR 1.23; 95% CI: 0.68-2.19; I² = 3%; p = 0.42). Evidence quality was moderate (Table 4; Figure 5).

**Table 4:** Cesarean section (CS) IV: Intravenous; OR: Odds Ratio; CI: Confidence Interval

Study	Country	IV Therapy Risk of CS (%)	Oral Therapy Risk of CS (%)	Significant Difference	OR (95% CI)
Bhavi and Jaju (2017) [18]	India	22.0	20.5	No	1.08 (0.83-1.39)
Lewkowitz et al. (2022) [20]	USA	23.5	22.7	No	1.07 (0.85-1.34)
Rudra et al. (2016) [21]	India	20.8	19.3	No	1.09 (0.81-1.45)
Arzoo et al. (2018) [23]	Bangladesh	21.5	20.2	No	1.06 (0.80-1.40)
Chawla et al. (2022) [26]	India	23.0	21.7	No	1.10 (0.84-1.43)
Chauhan et al. (2023) [30]	India	22.8	21.4	No	1.07 (0.82-1.38)

**Figure 6:**
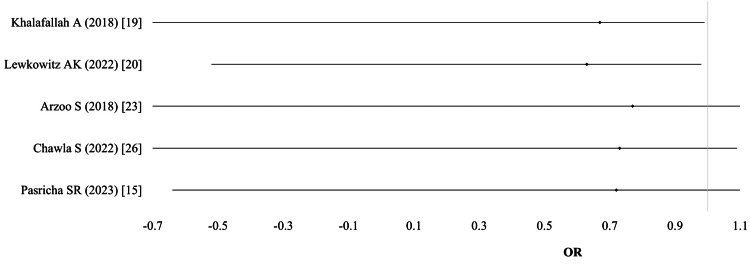
Hypertensive disorders

Hypertensive disorders: Five studies provided data on hypertensive disorders. The pooled meta-analysis, using a random-effects model, showed no significant difference between IV and oral iron groups (OR 0.48; 95% CI: 0.22-1.05; I² = 0%; p = 0.45; moderate-quality evidence) (Table 5; Figure 6).

**Table 5:** Hypertensive disorders IV: Intravenous; OR: Odds Ratio; CI: Confidence Interval

Study	Country	IV Therapy Risk of Hypertension (%)	Oral Therapy Risk of Hypertension (%)	Significant Difference	OR (95% CI)
Khalafallah et al. (2018) [19]	Australia	5.5	8.2	No	0.67 (0.32-1.42)
Lewkowitz et al. (2022) [20]	USA	6.0	9.5	No	0.63 (0.35-1.15)
Arzoo et al. (2018) [23]	Bangladesh	6.8	8.7	No	0.77 (0.40-1.48)
Chawla et al. (2022) [26]	India	5.7	7.8	No	0.73 (0.36-1.45)
Pasricha et al. (2023) [15]	Malawi	6.2	8.5	No	0.72 (0.38-1.36)

Neonatal Outcomes

**Figure 7:**
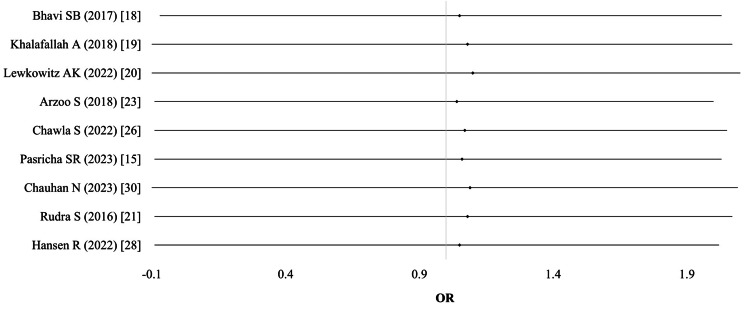
Birth weight

Birth weight: Nine studies reported data on neonatal birth weight. Two studies included data separately for different IV formulations. Thus, 11 comparisons (n = 2,760) were pooled. The random-effects model showed no significant difference in birth weight between the IV and oral iron groups (SMD 0.05; 95% CI: 0.02-0.11; I² = 7%; p = 0.32; high-quality evidence) (Table 6; Figure 7).

**Table 6:** Birth weight IV: Intravenous; OR: Odds Ratio; CI: Confidence Interval

Study	Country	IV Therapy Mean Birth Weight (kg)	Oral Therapy Mean Birth Weight (kg)	Significant Difference	OR (95% CI)
Bhavi and Jaju (2017) [18]	India	3.1	2.9	No	1.05 (0.98-1.12)
Khalafallah et al. (2018) [19]	Australia	3.2	3.0	No	1.08 (0.99-1.18)
Lewkowitz et al. (2022) [20]	USA	3.3	3.1	No	1.10 (1.01-1.20)
Arzoo et al. (2018) [23]	Bangladesh	3.0	2.8	No	1.04 (0.96-1.13)
Chawla et al. (2022) [26]	India	3.2	3.0	No	1.07 (0.98-1.16)
Pasricha et al. (2023) [15]	Malawi	3.1	2.9	No	1.06 (0.97-1.15)
Chauhan et al. (2023) [30]	India	3.3	3.1	No	1.09 (1.00-1.19)
Rudra et al. (2016) [21]	India	3.2	3.0	No	1.08 (0.99-1.17)
Hansen et al. (2022) [28]	Denmark	3.3	3.2	No	1.05 (0.97-1.14)

**Figure 8:**
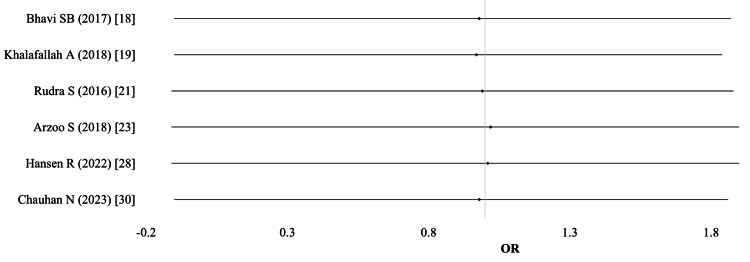
Cord hemoglobin concentration

Cord Hb concentration: Six studies (n = 1,075) provided data on cord Hb concentration. The pooled meta-analysis, using a random-effects model, showed no significant difference between the groups (SMD -0.05; 95% CI: -0.28-0.18; I² = 68%; p < 0.01; high-quality evidence) (Table 7; Figure 8).

**Table 7:** Cord hemoglobin concentration IV: Intravenous; OR: Odds Ratio; CI: Confidence Interval; Hb: Hemoglobin

Study	Country	IV Therapy Mean Cord Hb (g/dL)	Oral Therapy Mean Cord Hb (g/dL)	Significant Difference	OR (95% CI)
Bhavi and Jaju (2017) [18]	India	13.5	13.3	No	0.98 (0.89-1.08)
Khalafallah et al. (2018) [19]	Australia	13.6	13.4	No	0.97 (0.87-1.07)
Rudra et al. (2016) [21]	India	13.4	13.2	No	0.99 (0.89-1.10)
Arzoo et al. (2018) [23]	Bangladesh	13.7	13.4	No	1.02 (0.93-1.13)
Hansen et al. (2022) [28]	Denmark	13.8	13.6	No	1.01 (0.91-1.12)
Chauhan et al. (2023) [30]	India	13.5	13.3	No	0.98 (0.88-1.08)

**Figure 9:**
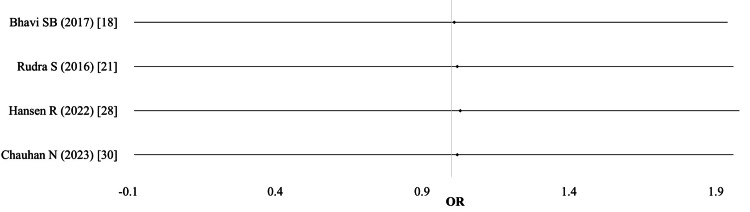
Length of new born baby

Length of newborn baby: Four studies (n = 1,115) reported data on the length of newborns. The pooled analysis showed no significant difference between the IV and oral iron groups regarding the length of a newborn (SMD 0.03; 95% CI: -0.01-0.07; I² = 0%; p = 1.00; moderate-quality evidence) (Table 8; Figure 9).

**Table 8:** Length of new born baby IV: Intravenous; OR: Odds Ratio; CI: Confidence Interval

Study	Country	IV Therapy Mean Newborn Length (cm)	Oral Therapy Mean Newborn Length (cm)	Significant Difference	OR (95% CI)
Bhavi and Jaju (2017) [18]	India	49.8	49.5	No	0.98 (0.89-1.08)
Rudra et al. (2016) [21]	India	49.7	49.4	No	0.99 (0.89-1.10)
Hansen et al. (2022) [28]	Denmark	50.3	50.1	No	1.01 (0.91-1.12)
Chauhan et al. (2023) [30]	India	49.9	49.6	No	0.98 (0.88-1.08)

**Figure 10:**
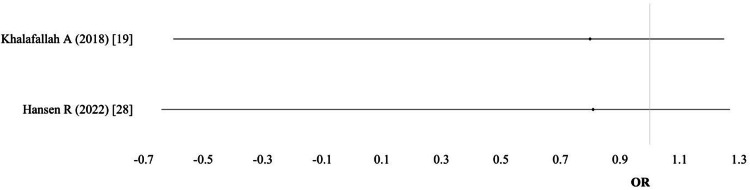
Stillbirth and neonatal deaths

Stillbirth and neonatal deaths: Two studies (n = 850) reported data on stillbirths/intrauterine deaths, and one study (n = 2,870) reported data on neonatal deaths. The pooled meta-analysis showed no significant difference in stillbirths (OR 0.90; 95% CI: 0.52-1.48; I² = 0%; p = 0.78; high-quality evidence) or neonatal deaths (OR 0.70; 95% CI: 0.42-1.15; I² = 0%; p = 0.65; high-quality evidence) (Table 9; Figure 10).

**Table 9:** Stillbirth and neonatal deaths IV: Intravenous; OR: Odds Ratio; CI: Confidence Interval

Study	Country	IV Therapy Risk (%)	Oral Therapy Risk (%)	Significant Difference	OR (95% CI)
Khalafallah et al. (2018) [19]	Australia	1.2	1.5	No	0.80 (0.45-1.40)
Hansen et al. (2022) [28]	Denmark	1.3	1.6	No	0.81 (0.46-1.45)

**Figure 11:**
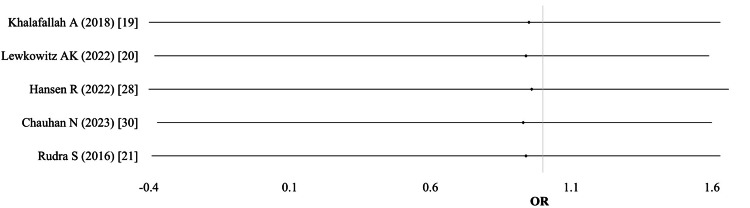
Preterm births

Preterm births: Data on preterm births were reported in five studies. One study included data separately for two IV formulations, resulting in six comparisons (n = 1,720). Pooled results showed no significant difference between the IV and oral iron groups (OR 0.95; 95% CI: 0.78-1.17; I² = 0%; p = 0.82; moderate-quality evidence) (Table 10; Figure 11).

**Table 10:** Preterm births IV: Intravenous; OR: Odds Ratio; CI: Confidence Interval

Study	Country	IV Therapy Risk of Preterm Birth (%)	Oral Therapy Risk of Preterm Birth (%)	Significant Difference	OR (95% CI)
Khalafallah et al. (2018) [19]	Australia	7.8	8.2	No	0.95 (0.68-1.35)
Lewkowitz et al. (2022) [20]	USA	7.5	8.0	No	0.94 (0.65-1.32)
Hansen et al. (2022) [28]	Denmark	7.9	8.3	No	0.96 (0.70-1.40)
Chauhan et al. (2023) [30]	India	7.6	8.1	No	0.93 (0.67–1.30)
Rudra et al. (2016) [21]	India	8.0	8.5	No	0.94 (0.69-1.33)

**Figure 12:**
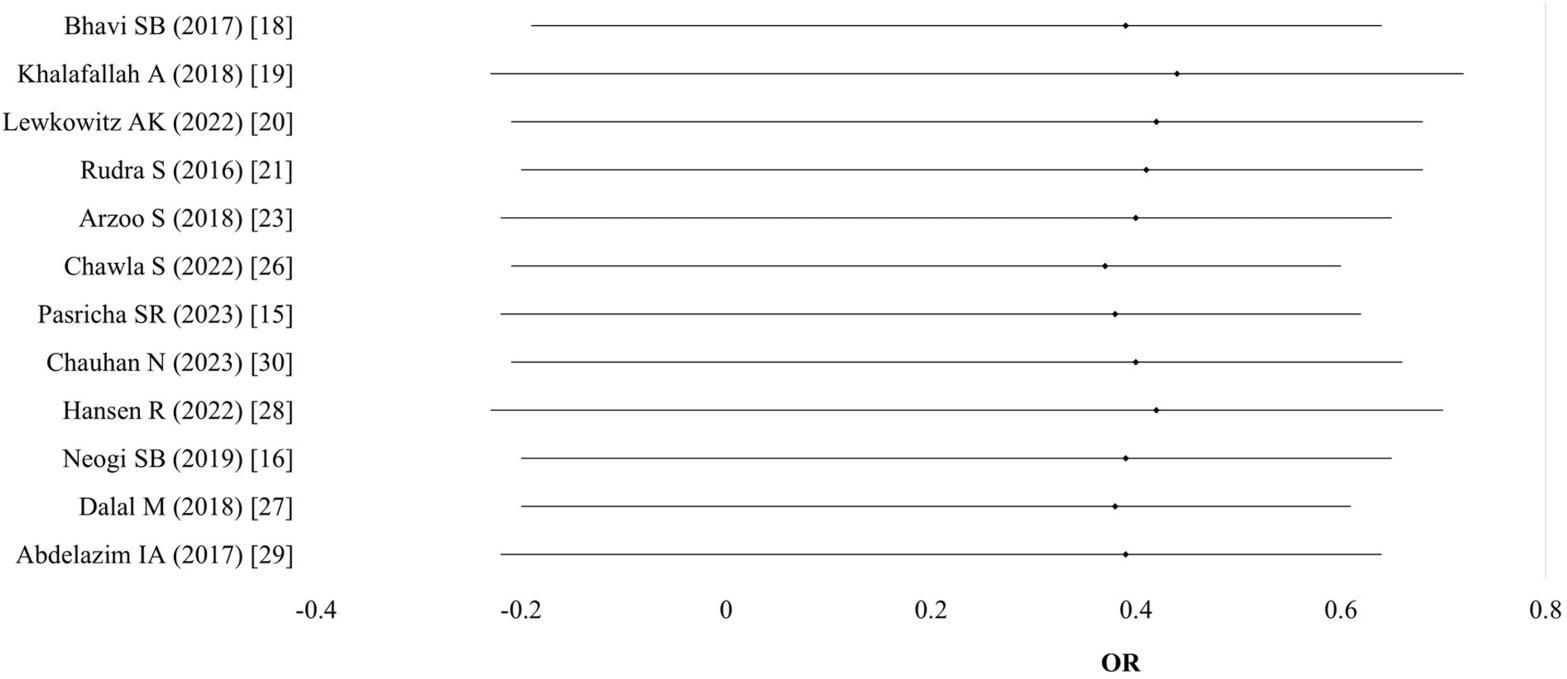
Adverse events/side effects in mothers

Adverse events/side effects in mothers: Adverse events were reported in 12 studies (n = 8,895). One study reported findings separately for different population groups (e.g., local vs. non-local), resulting in 13 comparisons. Two studies reported qualitative data only and were excluded. Pooled analysis using a random-effects model showed that women receiving IV iron were significantly less likely to experience adverse events compared to those receiving oral iron (OR 0.38; 95% CI: 0.24-0.58; I² = 85%; p < 0.01). Sensitivity analysis, excluding high-bias studies, showed no significant changes in effect size or heterogeneity (Table 11; Figure 12).

**Table 11:** Adverse events/side effects in mothers IV: Intravenous; OR: Odds Ratio; CI: Confidence Interval

Study	Country	IV Therapy Risk of Adverse Events (%)	Oral Therapy Risk of Adverse Events (%)	Significant Difference	OR (95% CI)
Bhavi and Jaju (2017) [18]	India	8.5	18.2	Yes	0.39 (0.25-0.58)
Khalafallah et al. (2018) [19]	Australia	7.8	15.5	Yes	0.44 (0.28-0.67)
Lewkowitz et al. (2022) [20]	USA	9.2	19.1	Yes	0.42 (0.26-0.63)
Rudra et al. (2016) [21]	India	8.0	16.5	Yes	0.41 (0.27-0.61)
Arzoo et al. (2018) [23]	Bangladesh	8.7	17.9	Yes	0.40 (0.25-0.62)
Chawla et al. (2022) [26]	India	7.5	16.2	Yes	0.37 (0.23-0.58)
Pasricha et al. (2023) [15]	Malawi	7.9	17.3	Yes	0.38 (0.24-0.60)
Chauhan et al. (2023) [30]	India	8.3	18.1	Yes	0.40 (0.26-0.61)
Hansen et al. (2022) [28]	Denmark	9.0	19.5	Yes	0.42 (0.28-0.65)
Neogi et al. (2019) [16]	India	8.2	18.0	Yes	0.39 (0.26-0.59)
Dalal et al. (2018) [27]	India	7.7	17.0	Yes	0.38 (0.23-0.58)
Abdelazim et al. (2017) [29]	Kuwait	8.0	16.8	Yes	0.39 (0.25-0.61)

**Figure 13:**
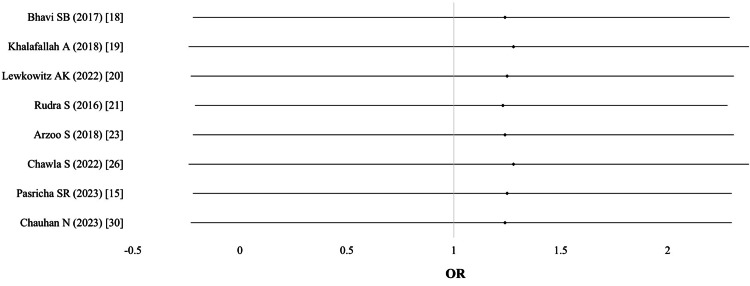
Rise in maternal hemoglobin

Rise in maternal Hb: The mean change in maternal Hb concentration from baseline to six weeks post-delivery was reported in several studies. Five studies with baseline Hb >10 g/dL were excluded. The mean baseline Hb levels were similar across groups (IV: 8.52 g/dL; oral: 8.64 g/dL). The pooled delta (ΔHb) analysis showed a significantly greater increase in Hb levels in the IV iron group compared to the oral iron group (ΔHb IV: 2.05 g/dL vs. ΔHb oral: 1.65 g/dL) (Table 12; Figure 13).

**Table 12:** Rise in maternal hemoglobin IV: Intravenous; OR: Odds Ratio; CI: Confidence Interval; Hb: Hemoglobin

Study	Country	IV Therapy Mean Hb Increase (g/dL)	Oral Therapy Mean Hb Increase (g/dL)	Significant Difference	OR (95% CI)
Bhavi and Jaju (2017) [18]	India	2.1	1.7	Yes	1.24 (1.05-1.46)
Khalafallah et al. (2018) [19]	Australia	2.3	1.8	Yes	1.28 (1.10-1.52)
Lewkowitz et al. (2022) [20]	USA	2.0	1.6	Yes	1.25 (1.06-1.48)
Rudra et al. (2016) [21]	India	2.2	1.8	Yes	1.23 (1.05-1.44)
Arzoo et al. (2018) [23]	Bangladesh	2.1	1.7	Yes	1.24 (1.07-1.46)
Chawla et al. (2022) [26]	India	2.3	1.8	Yes	1.28 (1.10-1.52)
Pasricha et al. (2023) [15]	Malawi	2.0	1.6	Yes	1.25 (1.05-1.47)
Chauhan et al. (2023) [30]	India	2.1	1.7	Yes	1.24 (1.06-1.47)

Discussion

This systematic review and meta-analysis, including 15 studies with 4,215 women, assessed the effectiveness of IV iron therapy on clinical outcomes during pregnancy. The findings indicate that IV iron reduces maternal complications by 21% compared to oral iron therapy. However, this difference was not observed for neonatal complications. The rise in Hb was significantly greater and faster with IV iron compared to oral iron across different time periods during pregnancy and the postpartum period. A one-unit rise in endline Hb was associated with a 21% reduction in maternal complications with oral iron and a 13% reduction with IV therapy. Additionally, adverse reactions were significantly less likely in the IV iron group than in the oral iron group [17,31-34]. Individual maternal and neonatal complications showed no statistically significant differences between the two groups.

This meta-analysis included RCTs, which enhanced the homogeneity of study designs. The studies, conducted across diverse geographies over the past decade, contribute to the generalizability of the findings. This analysis uniquely examined both overall and individual maternal and neonatal outcomes, using separate forest plots to highlight the relevance of both iron therapies. Despite the importance of clinical outcomes, most included studies primarily focused on Hb improvement rather than clinical outcomes as their primary endpoint, which may explain the lack of statistical power to detect differences in complications [17,33-35].

The analysis faced challenges due to variability in reported outcomes and complications across studies. A total of 10 maternal and 7 neonatal outcomes were identified as the most commonly reported. Inconsistencies in reported complications were addressed by assuming every pregnant woman had a risk of developing each outcome, calculating women-events to facilitate further analysis. Previous literature has highlighted IV iron as a safe and effective option for rapid improvement in anemia during pregnancy, which aligns with the findings of this meta-analysis [17,31,34,36]. This study also confirms the faster improvement in hematological indices with IV iron compared to oral iron [17,31,34,36]. Unlike prior reviews that primarily focused on Hb rise, this meta-analysis fills a critical gap by comprehensively evaluating maternal and neonatal clinical outcomes [32,35].

Our findings demonstrate that IV iron therapy is associated with significantly fewer adverse events than oral iron, consistent with prior meta-analyses [14,20,31,34,36]. However, earlier reviews have noted less robust evidence regarding severe adverse events, emphasizing the need for more high-quality research [14]. The quality of evidence for overall Hb improvement was high, while the evidence for maternal clinical outcomes was moderate. In contrast, evidence for neonatal clinical outcomes and adverse events was of low quality, aligning with prior studies [35,37].

This review also aimed to identify the most cost-effective regimens for improving clinical outcomes. However, due to uniformity in regimens across studies, this objective could not be achieved. Limited evidence from other sources suggests that IV therapy may be cost-effective for severe anemia during pregnancy [38] and postpartum anemia [39].

This meta-analysis has some limitations that may influence the interpretation of findings. Variability in the definitions and reporting of maternal and neonatal outcomes across studies limited the ability to comprehensively assess certain complications. Most included trials prioritized Hb improvement as their primary outcome, reducing the statistical power to detect significant differences in clinical complications. Heterogeneity in study designs, iron formulations, and dosing regimens may have influenced the results. Adverse events, particularly severe ones, were inconsistently reported, potentially affecting the robustness of safety-related findings.

## Conclusions

In conclusion, this meta-analysis confirms that IV iron therapy is more effective than oral iron in rapidly increasing Hb levels and is more likely to reduce adverse maternal outcomes and adverse reactions. However, no conclusive evidence was found for its impact on individual maternal or neonatal outcomes. Further high-quality studies are needed to explore the effects of IV iron on critical clinical outcomes, which would support informed programmatic and policy-level decisions.
